# Comprehensive Immune Profiling of a Kidney Transplant Recipient With Peri-Operative SARS-CoV-2 Infection: A Case Report

**DOI:** 10.3389/fimmu.2021.753558

**Published:** 2021-09-22

**Authors:** Karen R. Sherwood, David D. M. Nicholl, Franz Fenninger, Vivian Wu, Paaksum Wong, Vince Benedicto, Davide P. Cina, Meng Wang, Taylor D. Pobran, Mari L. De Marco, Anna Citlali Márquez, Agatha N. Jassem, Inna Sekirov, Muhammad G. Morshed, Mohammad Bardi, Mypinder Sekhon, Paul Keown, Matthew Kadatz, James H. Lan

**Affiliations:** ^1^Department of Medicine, University of British Columbia, Vancouver, BC, Canada; ^2^Department of Pathology and Laboratory Medicine, University of British Columbia, Vancouver, BC, Canada; ^3^Division of Nephrology, University of British Columbia, Vancouver, BC, Canada; ^4^British Columbia Provincial Immunology Laboratory, University of British Columbia, Vancouver, BC, Canada; ^5^Department of Urologic Sciences, University of British Columbia, Vancouver, BC, Canada; ^6^Department of Pathology and Laboratory Medicine, Providence Health Care, Vancouver, BC, Canada; ^7^British Columbia Center for Disease Control Public Health Laboratory, Provincial Health Services Authority, Vancouver, BC, Canada; ^8^Division of Rheumatology, University of British Columbia, Vancouver, BC, Canada

**Keywords:** COVID-19, transplant, induction therapy, immunosuppressants, immune response

## Abstract

To date there is limited data on the immune profile and outcomes of solid organ transplant recipients who encounter COVID-19 infection early post-transplant. Here we present a unique case where the kidney recipient’s transplant surgery coincided with a positive SARS-CoV-2 test and the patient subsequently developed symptomatic COVID-19 perioperatively. We performed comprehensive immunological monitoring of cellular, proteomic, and serological changes during the first 4 critical months post-infection. We showed that continuation of basiliximab induction and maintenance of triple immunosuppression did not significantly impair the host’s ability to mount a robust immune response against symptomatic COVID-19 infection diagnosed within the first week post-transplant.

## Introduction

**Figure 1:**
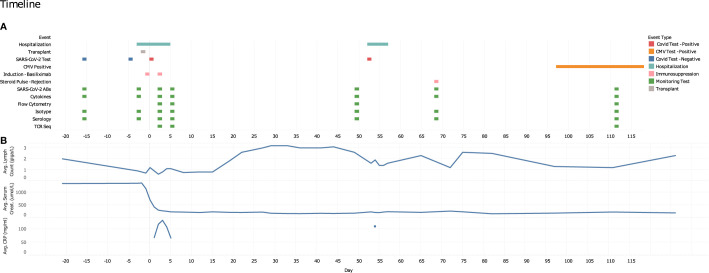
Study time points and data collection points. **(A)** Graphical representation of pertinent clinical data, sample collection time points and immune monitoring testing. **(B)** CBC differentials (lymphocyte count, giga/L), average serum creatinine levels (μmol/L) and serum C-reactive protein levels (mg/ml) over the course of the patient’s transplant and infection disease course (days).

A robust immune response is critical to achieving full recovery from COVID-19 infection. Kidney transplant recipients undergo T cell depleting induction therapy followed by maintenance immunosuppression which alters the viral response. There is limited data on the immune response and outcomes of kidney transplant recipients who develop COVID-19 in the early post-transplant period after having undergone induction therapy. Here we present a comprehensive longitudinal assessment of the immune response in a renal transplant recipient who developed symptomatic COVID-19 perioperatively. Our evaluation includes SARS-CoV-2 serology, serum antibody levels, immunoglobulin isotype quantification, T cell receptor sequencing, serum cytokine profiling, and cytometric immune cell profiling (**Figure 1**).

## Case Description

A 30-year-old male with IgA nephropathy received a living-donor kidney transplant at Vancouver General Hospital, Canada. Pre-transplant flow-cytometric crossmatch and donor-specific antibody testing were both negative. Prior to surgery, both recipient and donor self-isolated for 2 weeks. Nasopharyngeal swabs for COVID-19 were performed on both donor and recipient at the beginning and end of their self-isolation with all results were negative. The patient received basiliximab on day 0 and 4, and was maintained on prednisone, tacrolimus, and mycophenolate mofetil thereafter. Some hours after the surgery, the patient was informed of a COVID-19 exposure and tested positive on postoperative day 2. He became symptomatic with cough, dyspnea on exertion, fevers, and night sweats. He remained clinically well enough however to be discharged on post-operative day 7. During his hospitalization, serial inflammatory markers were measured and showed an elevation in ferritin (peak 1565 ug/L), C-reactive protein (peak 133 mg/mL), d-dimer (peaked 960 ug/L), and fibrinogen (peak 7.9 g/L). His COVID-19 symptoms fully resolved after 1 month. His post-transplant course was also complicated by borderline acute cellular rejection at week 10 that was treated with pulse methylprednisolone, and CMV viremia between weeks 14 and 20 post-transplant. Written informed consent was obtained from the patient for this report.

## Diagnostic Investigation

### SARS-CoV-2 Serology, Antibody Reactivities and Immunoglobulin Subclass Dynamics Were Consistent With Mild Infection Even in the Context of Induction Therapy

**Figure 2:**
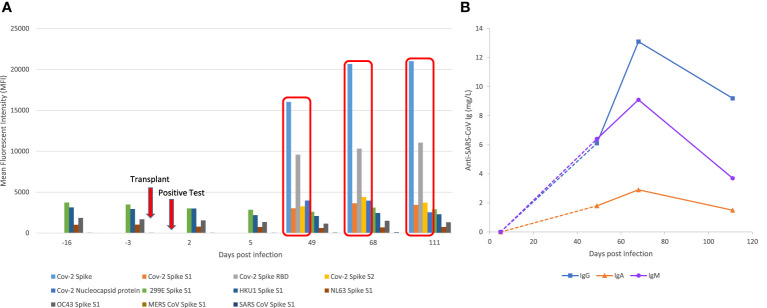
SARS-CoV2 and non-CoV-2 antibody profiling using Luminex based assays and immunoglobulin subclass assays. **(A)** Reactivity of serum IgG antibody to CoV-2 proteins (Full Spike, S1, S2, RBD and NC) and non-SARS-CoV-2 proteins from community coronaviruses (CoV-229E-S1, HCoV-HKU1-S1, HCoV-NL63-S1, HCoV-OC43-S1), and novel coronaviruses (MERS-S1 and SARS-S1). X-axis represents days relative to first positive SARS-CoV-2 test in chronological serum dates. Y-axis represents the Mean Florescence Intensity (MFI) value of the reactivity. **(B)** Anti-SARS-CoV-2 IgG, IgA and IgM serum concentrations measured using the immunIQ assay (mg/L).

Evaluation of serum antibodies was performed using a semi-quantitative multiplexed Luminex bead-based assay, which detects IgG antibodies to SARS-CoV-2 proteins: full spike, spike 1 (S1), spike 2 (S2), RBD, and NC protein. This assay also detects antibodies to proteins from endemic human (CoV-229E-S1, HCoV-HKU1-S1, HCoV-NL63-S1, HCoV-OC43-S1) and novel coronaviruses (MERS-S1 and SARS-S1). Anti-SARS-CoV-2 serologies were further evaluated using MesoScale Discovery V-Plex, a multiplex chemiluminescent immunoassay targeting both SARS-CoV-2 and endemic human coronavirus targets that have been validated against platforms in clinical use (data not shown). No SARS-CoV-2 specific antibodies were detected in samples prior to day 49 by either assay (**Figure 2A**). Low levels of serum IgG antibodies (MFI <4,000) to community coronaviruses were detected prior to the first positive SARS-CoV-2 test, suggesting previous exposure to other coronaviruses (**Figure 2A**). From day 49 onwards, MFI values for antibodies specific to CoV-2 spike and RBD proteins increased significantly (MFI > 10,000); MFI values for nucleocapsid and S2 protein were also increased (MFI >3,500). To further characterize the humoral response, we performed an in-house ImmunIQ assay which quantitatively detects anti-SARS-CoV-2 IgG, IgA, and IgM isotypes by using the RBD domain from the spike protein as the capture antigen. The concentration of IgM, IgG and IgA were below detectable limits in post-op day 5 samples. Increasing levels of all three isotypes were detected from day 49, with peak concentrations in day 68 samples (IgG 13.1, IgM 9.1, IgA 2.9 mg/L) (**Figure 2B**). The patient’s post-transplant anti-HLA antibody testing showed no *de novo* donor-specific antibodies.

### Cytokine Profiling Shows Limited Evidence of an Inflammatory Phenotype

21 cytokines commonly associated with the previously described COVID-19 ‘cytokine storm’ phenomenon (1–4) were evaluated using the ProCartaPlex Luminex panel. Of these, 12 were below the limits of detection. Only 8 cytokines were detected consistently across all tested longitudinal samples, and of these, levels of IL-18, IP-10, MCP-1, and MIP-1β were all below normal reference values (**Supplementary Table 1**) (5, 6).

### Anti-SARS-CoV-2 T Cell Repertoire Analysis Confirms Previous Exposure to Community Coronaviruses and Limited SARS-CoV-2 Specific Changes

**Figure 3:**
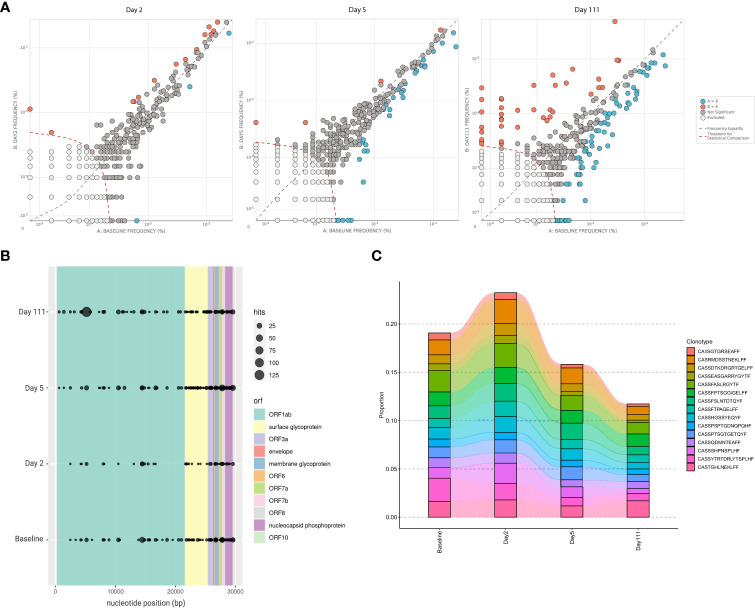
T cell repertoire sequencing of patient at study time points. **(A)** Differential abundance analysis comparing clonotypes that have significantly increased or decreased in frequency across baseline and post-infection Day 2, Day 5, and Day 111. This is based on a beta-binomial model where normal repertoire changes in healthy adults over time are accounted for while measuring clonal expansion. **(B)** Locations on the SARS-CoV-2 genome where TCR binding has likely occurring; different colours specify open reading frame, and size of dots indicate number of TCRβ rearrangements associated with SARS-CoV-2 at a particular position. Screening was performed based on the ImmuneCODE database (Adaptive Biotechnologies). **(C)** Changes in frequencies of the 15 most frequently observed TCR clonotypes, relative to total TCR repertoire, across baseline and post-infection day 2, 5, and 111.

Peripheral T cell repertoire profiling was performed for human TCRβ genes on a baseline sample taken prior to the SARS-CoV-2 epidemic, and multiple time points post COVID-19 infection. Differential abundance analysis using beta-binomial modelling to account for variability over time, showed limited differences in overall clonal expansion between the baseline sample, and either day 2 or day 5 samples (7). By day 111 however, there were a number of public clonotypes which increased in frequency compared to baseline (**Figure 3A**). Mapping these clonotypes against the ImmuneCODE database, which contains reference data for SARS-CoV-2-associated TCRs, revealed the presence of multiple public TCRs targeting different regions of the SARS-CoV-2 genome (**Figure 3B**). The evidence of such clonotypes in the baseline sample suggests previous exposure to community coronaviruses, with multiple targets mapping to the ORF1 and surface glycoprotein, both regions of which are conserved across various coronaviridae genomes (**Figure 3B**). The top 15 occurring clonotypes across all samples did not show demonstrable changes in the repertoire over the course of infection (**Figure 3C**).

### Cellular Immune Profiling Showed Minimal Demonstrable T Cell Changes but More Significant Alterations in B Cell Subsets

**Figure 4:**
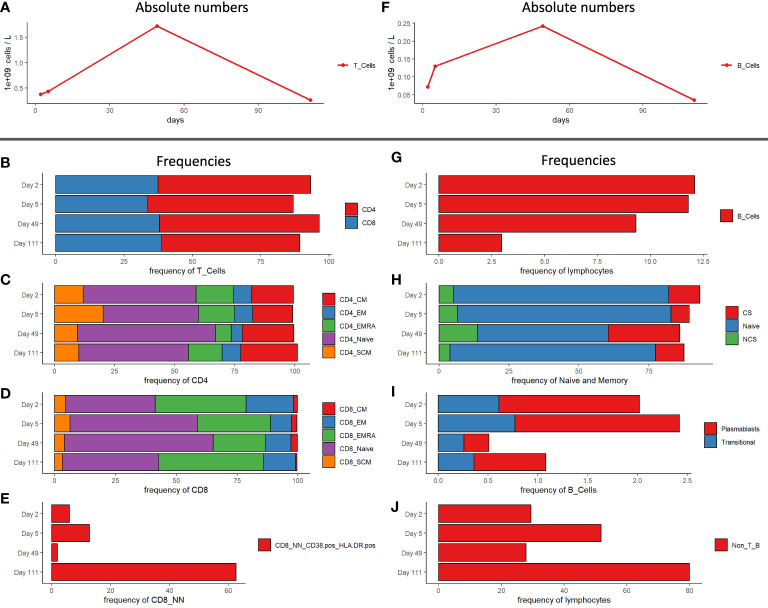
Immunophenotyping of PBMCs of patient at study time points. **(A)** Absolute numbers of T cells. **(B)** CD4 and CD8 T cell frequencies. **(C)** Subpopulations frequencies of CD4 T cells. **(D)** Subpopulation frequencies of CD8 T cells. **(E)** CD38^+^ HLA-DR^+^ frequencies of CD8 non-naïve T cells. **(F)** Absolute numbers of B cells. **(G)** B cell frequencies of lymphocytes. **(H)** Class switched -, non-class switched – and naïve B cell frequencies of naïve and memory B cells. **(I)** Plasmablast - and transitional B cell frequencies of total B cells. **(J)** Non-T- and Non-B-cell frequencies of lymphocytes. CM, central memory; EM, effector memory; SCM, stem cell memory; CS, class-switched; NCS, non class-switched; Non_T_B, non-T, and non-B cells.

Immune cell phenotyping from whole blood was performed for T regulatory, T memory and B cells. The patient was T cell lymphopenic post-transplant and during the early phase of infection as evident in the immunophenotypic profile on day 2 and 5 (0.37 and 0.43 x 10^9^ cells/L) post-infection (**Figure 4A**). By day 49 (1.72 x 10^9^ cells/L), T cell counts had returned to within normal range, however the patient was again lymphopenic by day 111 (0.26 x 10^9^ cells/L) (**Figure 4A**). CD4 (50.5 – 58.4%) and CD8 T cell frequencies (33.7 – 38.8%) remained unchanged during the study period (**Figure 4B**) with only minor changes observed in CD4 T cell subsets (**Figure 4C**). Alterations in CD8 T cell compartments were more pronounced compared to CD4 compartments as would be expected in the context of viral infection. The frequency of CD8 EMRA T cells was greatest during the acute phase of infection on day 2 (37.4%) and day 5 (30.1%), then decreased on day 49 (21.5%). By day 111 the frequency of CD8 EMRA T cells rose again to day 2 levels (43.1%). In contrast, naïve CD8 T cells frequencies were decreased on day 2 and 5 (36.8 and 52.4% respectively), expanded on day 49 (61.1%), then contracted by day 111 (39.5%) (**Figure 4D**). The frequency of activated non-naïve CD8 T cells (CD38+ HLA-DR+) was significantly increased on day 111 (62.7%), compared to the earlier time points (1.92 – 12.8%) (**Figure 4E**). Like T cell subsets, B cell absolute counts were generally low on day 2 and 5 (0.07 and 0.13 x 10^9^ cells/L respectively), recovered to normal by day 49 (0.24 x 10^9^ cells/L), then diminished again by day 111 (0.03 x 10^9^ cells/L blood) (**Figure 4F**). The frequency of B cells, as a proportion of lymphocytes, on the other hand dropped steadily (12.1, 11.8, 9.3 and 3%) over the observation period (**Figure 4G**). The absolute counts of B cell subsets were low in the early phase of infection and the frequency of these subsets including naïve, class-switched, and non-class switched memory B cells changed only slightly (**Figure 4H**). The frequency of plasmablasts was elevated on days 2 and 5 (1.41 and 1.65% respectively), then declined at day 49 (0.25%), and increased again by day 111 (0.72%) (**Figure 4I**). Finally, the Non-T- and Non-B-cell frequency of lymphocytes increased from 29.4 on day 2 to 51.8% on day 5. Subsequently it declined to 27.9 (day 49) before reaching 80% on day 111 (**Figure 4J**).

## Discussion

Optimal immunosuppression management in kidney transplant recipients with COVID-19 remains an open clinical question. The concern for allograft rejection must be balanced with permissive immunocompetence to allow for patients to generate a robust antiviral immune response. In this report, we present the unique case of a kidney transplant recipient who contracted COVID-19 in the perioperative window during which he received induction therapy.

SARS-CoV-2 antibodies and immunoglobulin subclass dynamics were in keeping with prior reports in which most patients infected with SARS-CoV-2 seroconverted within 14 days of exposure to *de novo* viral infection (8). Based on previously reported values, this patient’s IgG, IgM, and IgA response was more consistent with a mild response to infection (9, 10). Antibody isotype kinetics revealed a prolonged profile when compared to non-transplant COVID patients (11), which may be expected as shedding is often prolonged in kidney transplant patients (12). The observed humoral response was evident even in the context of induction therapy and immunosuppression. In contrast, there were limited cytokine changes over the course of infection. This non-inflammatory phenotype, whilst surprising, could be attributed to the highly dynamic nature of cytokine production which varies significantly depending on pre-existing medical conditions, demographic and environmental factors such as age or prior herpesvirus exposure (13). In addition, the demonstrable cytokine levels in this patient may be confounded by the use of immunosuppression. Cytokine levels have also been challenging to correlate with mild disease as only a subset of COVID-19 patients exhibit dramatic ‘cytokine storm’ phenotypes (2). Immunophenotyping showed changes in lymphocyte subsets throughout the patient’s illness and convalescence that were consistent with the findings of other groups in both the non-transplant and transplant setting (2, 14, 15), including the observed T cell lymphopenia. Subcompartment analysis showed an increased frequency of CD8 TEMRA and activated (CD38+ HLA-DR+) non-naive CD8 T cells during early infection, which is consistent with observations in a non-immune supressed cohort (16, 17). While only moderately altered, our observed T cell profile is consistent with a larger study of over 100 COVID-19 patients who had a mild course of their illness and recovered without requiring hospitalization (16). We also report an increased frequency of plasmablasts consistent with a B cell activation signature, consistent with other reports (3, 9, 10, 16). The appearance of this B cell signature correlates with detectable serum anti‐SARS‐CoV‐2 immunoglobulin and the resolution of the patient’s symptoms. One finding of interest is the late-stage re-expansion of CD8 EMRA, activated (CD38+ HLA-DR+) non-naive T cells, and plasmablasts on day 111. Our patient however tested positive for CMV viremia at week 14 which would be a more plausible explanation for this phenomenon, rather than the original COVID-19 infection, although ‘second wave’ responses have been reported (18).

Whether immunosuppression should be empirically reduced in transplant patients with a COVID-19 infection remains controversial (19, 20). In this instance, we chose to proceed with standard induction and maintenance immunosuppression due to the patient’s favourable clinical characteristics and his relatively mild COVID-19 course. Indeed, even with our effort to maintain standard immunosuppression, this patient developed borderline T cell rejection 2 months post-transplant. A retrospective HLA eplet analysis (HLAMatchmaker v2.0) showed that the patient had a high burden of eplet mismatches for acute rejection (HLA-DR sum mismatch of 18) which may explain this event. Fortunately, this was easily reversed with steroid therapy leading to resolution of his graft dysfunction. Interestingly, most of the immunophenotypic changes resembling an active SARS-CoV-2 infection were resolved by day 49, correlating with the timing of our patient’s clinical recovery. The recurrence of a similar immune signature on day 111 when the patient developed CMV viremia, the lymphocyte changes we observed with SARS-CoV-2 infection may not be specific, but rather represent a general adaptive response against viral pathogens. Given the susceptibility of kidney recipients to both viral and bacterial infections post-transplant, any longitudinal immunophenotype analysis of the immune response to COVID-19 needs to be interpreted in the context of other relevant clinical events.

In summary, in this unique case report, we showed that basiliximab induction therapy and maintenance of triple immunosuppression did not lsignificantly impair the recipient’s ability to mount a protective immune response against symptomatic COVID-19 infection occurring within the first week post-transplant. These results may not however be generalized to depleting induction agents such as anti-thymocyte globulin, which may have a different clinical impact. Longitudinal evaluation of immune parameters, together with recipient risk stratification for rejection, could represent a novel approach to guide optimal management of immunosuppressive agents in transplant patients with a COVID-19 infection.

## Data Availability Statement

The flow cytometric data presented in the study are deposited in the flowrepository database, accession number FR-FCM-Z4GA. The TCRSeq data presented in the study are deposited in the BioSample database (https://ncbi.nlm.nih.gov/biosample) repository, accession number SAMN21338671 and SAMN21338972.

## Ethics Statement

The studies involving human participants were reviewed and approved by University of British Columbia Clinical Research Ethics Committee. The patients/participants provided their written informed consent to participate in this study. Written informed consent was obtained from the individual(s) for the publication of any potentially identifiable images or data included in this article.

## Author Contributions 

KRS and JHL developed and funded the study. KRS and DN (specifically the clinical case) wrote the manuscript in consultation with DPC, FF, PW, MK, and JHL. FF, VW, PW, VB, MW, TDP, and CM designed and carried out the experiments. DPC, MD, AJ, IS, MM, MB, MS, PK, and MK provided feedback to the final manuscript. KRS wrote the manuscript and was in charge of overall direction, planning and supervision. All authors contributed to the article and approved the submitted version.

## Funding

This work was supported by funding to JHL from the Vancouver Coastal Health Research Institute 2020 COVID-19 Research fund, and to MLD from the Michael Smith Foundation for Health Research (16353; 2020-1199) and the Canadian Foundation for Innovation (40962).

## Conflict of Interest

The authors declare that the research was conducted in the absence of any commercial or financial relationships that could be construed as a potential conflict of interest.

## Publisher’s Note

All claims expressed in this article are solely those of the authors and do not necessarily represent those of their affiliated organizations, or those of the publisher, the editors and the reviewers. Any product that may be evaluated in this article, or claim that may be made by its manufacturer, is not guaranteed or endorsed by the publisher.
