# Supplementary material for: Comprehensive Immune Profiling of a Kidney Transplant Recipient With Peri-Operative SARS-CoV-2 Infection: A Case Report
Source: Front Immunol. 2021 Sep 22;12:753558. doi: 10.3389/fimmu.2021.753558 (PMC8492986; doi:10.3389/fimmu.2021.753558)

**Supplementary Table/Figure Legends:**

**Supplementary Table 1.** Serum cytokine concentrations as detected by ProcartaPlex Immunoassay, from pre-infection day -3 for duration of study period (post-infection day 111). Numbers reported as mean final concentrations (pg/ml). OOV<: Out Of Range.

**Supplementary Figure 1.** Cytokine profiling of detectable serum cytokines (mean concentrations pg/ml) over the clinical course of SARS-CoV-2 infection in case patient. Analytes which were out of range of detection are not shown here.

**Supplementary Figure 2.** Immunophenotyping of PBMCs of patient samples at study time points. a) Frequencies of T cell subsets. b) Frequencies of B cell subsets. Em: Effector memory. CS: Class-switched. NCS: Non-class switched. NN: Non-naïve. Non_T_B: Non T and Non B cells.

**Supplementary Figure 3.** a) Distribution of TRBV gene usage. b) CDR3 length by number of amino acids. c) Venn diagram showing number of amino acid overlaps in the CDR3 sequence between timepoint samples. d) Relative abundance of clonotypes with specific frequencies as a proportion of total TCR repertoire. e) Distribution of clonotype abundances. f) TCR repertoire similarity visualized in an overlap heatmap based on indices of i) number of shared public clonotypes and ii) Jaccard index.

| **Day post-infection** | **G-CSF (CSF-3)** | **GM-CSF** | **IFN alpha** | **IFN gamma** | **IL-1 beta** | **IL-2** | **IL-4** | **IL-5** | **IL-6** | **IL-8 (CXCL8)** | **IL-10** | **IL-12p70** | **IL-13** | **IL-17A (CTLA-8)** | **IL-18** | **IP-10 (CXCL10)** | **MCP-1 (CCL2)** | **MIP-1 alpha (CCL3)** | **MIP-1 beta (CCL4)** | **TNF alpha** | **TNF beta** |
| --- | --- | --- | --- | --- | --- | --- | --- | --- | --- | --- | --- | --- | --- | --- | --- | --- | --- | --- | --- | --- | --- |
| -3 | OOR< | OOR< | 2.39 | 5.98 | OOR< | OOR< | 24.25 | OOR< | OOR< | 15.81 | OOR< | OOR< | 2.17 | OOR< | 57.61 | 127.47 | 47.3 | 5.1 | 226.53 | OOR< | OOR< |
| 2 | 7.06 | OOR< | 5.885 | OOR< | OOR< | OOR< | 31.34 | OOR< | OOR< | OOR< | 1.05 | OOR< | 2.885 | OOR< | 71.805 | 80.975 | 33.62 | OOR< | 104.805 | OOR< | OOR< |
| 5 | 4.88 | OOR< | 3.01 | OOR< | OOR< | OOR< | 21.92 | OOR< | OOR< | OOR< | OOR< | OOR< | 2.885 | OOR< | 57.615 | 45.065 | 15.415 | OOR< | 54.505 | OOR< | OOR< |
| 49 | 4.88 | OOR< | OOR< | OOR< | OOR< | OOR< | OOR< | OOR< | OOR< | OOR< | OOR< | OOR< | OOR< | OOR< | 39.735 | 9.93 | 24.625 | OOR< | 96.235 | OOR< | OOR< |
| 68 | OOR< | OOR< | OOR< | 2.24 | OOR< | OOR< | OOR< | OOR< | OOR< | OOR< | OOR< | OOR< | 2.17 | OOR< | 11.36 | 14.95 | 18.22 | OOR< | 51.64 | OOR< | OOR< |
| 111 | OOR< | OOR< | OOR< | 2.21 | OOR< | OOR< | 22.72 | OOR< | OOR< | OOR< | 3.38 | OOR< | OOR< | OOR< | 234.2 | 23.74 | 23.41 | OOR< | 138.09 | OOR< | OOR< |


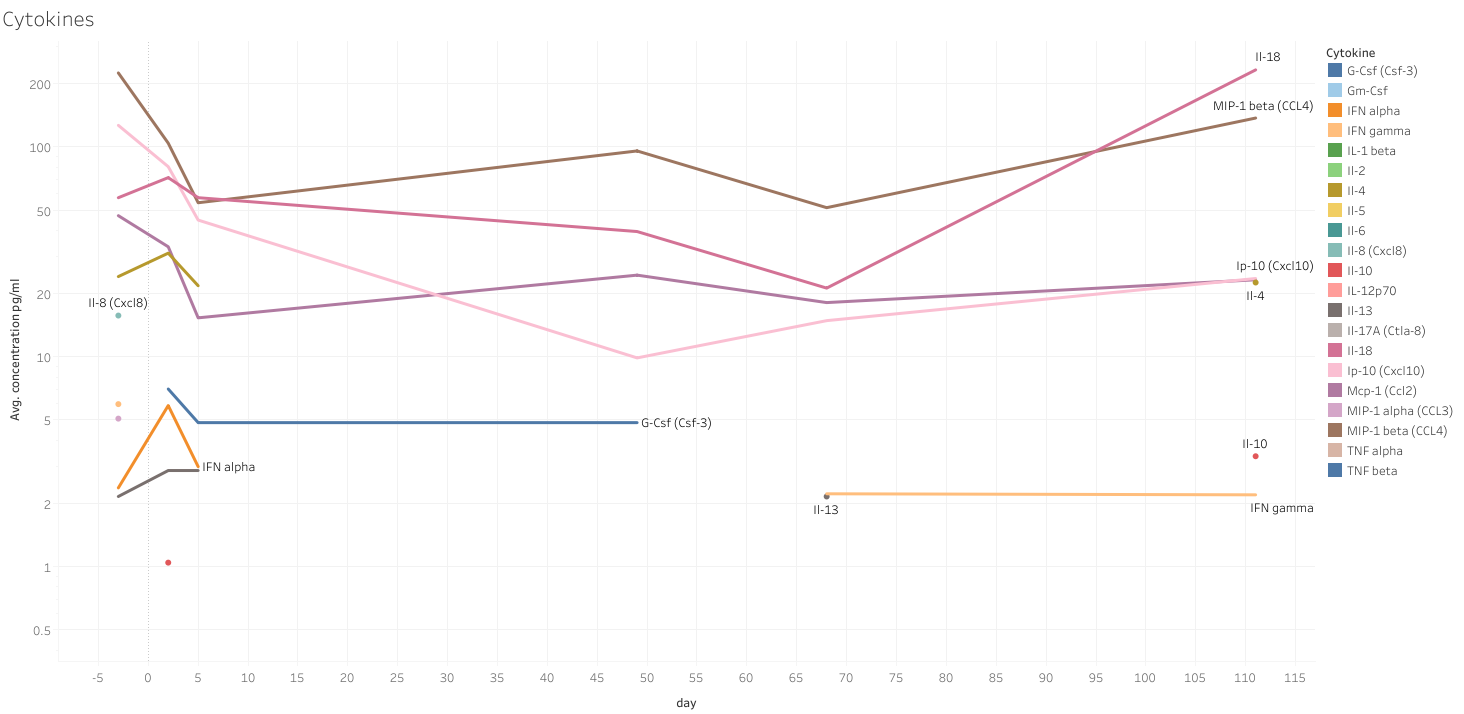


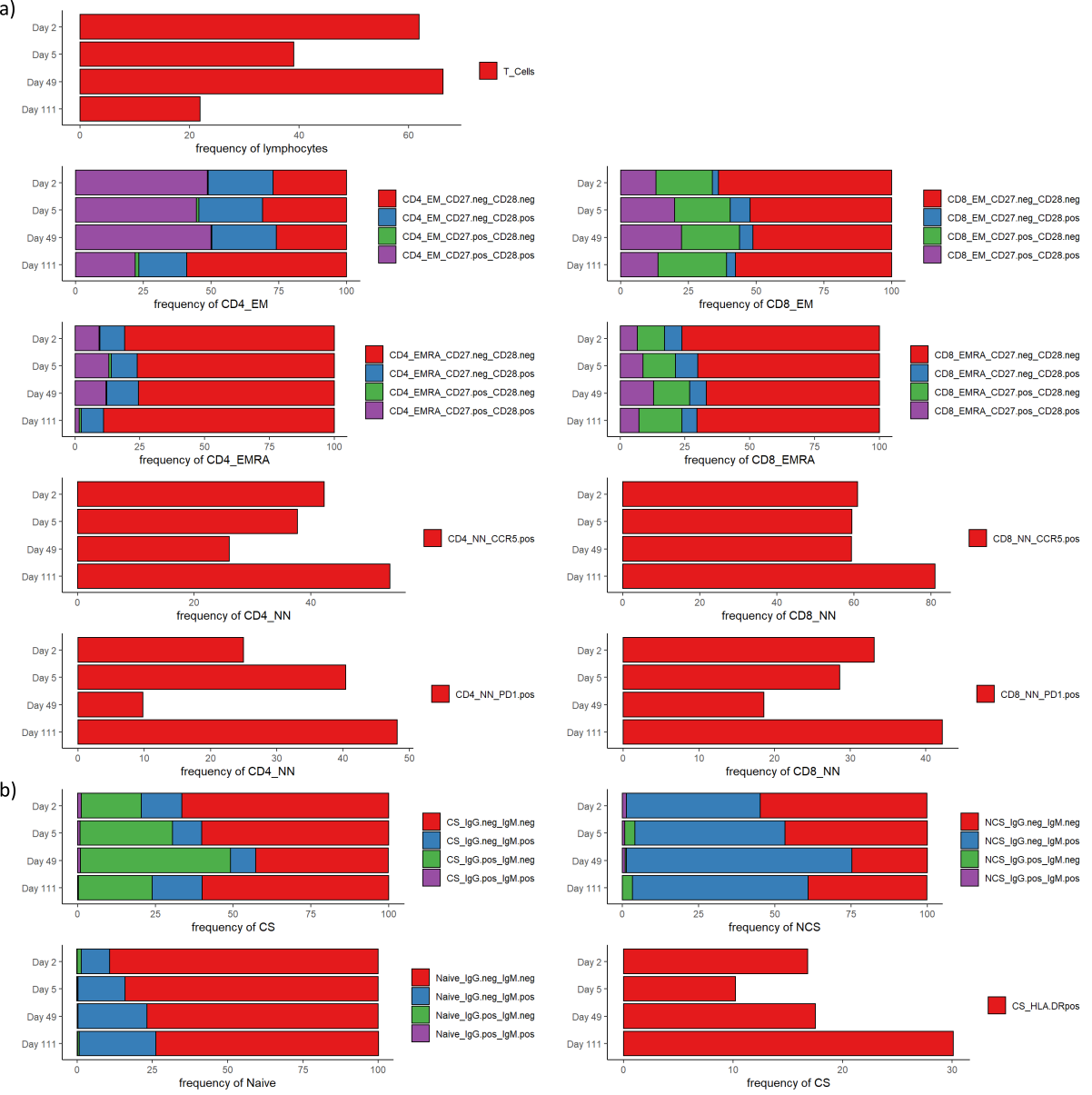

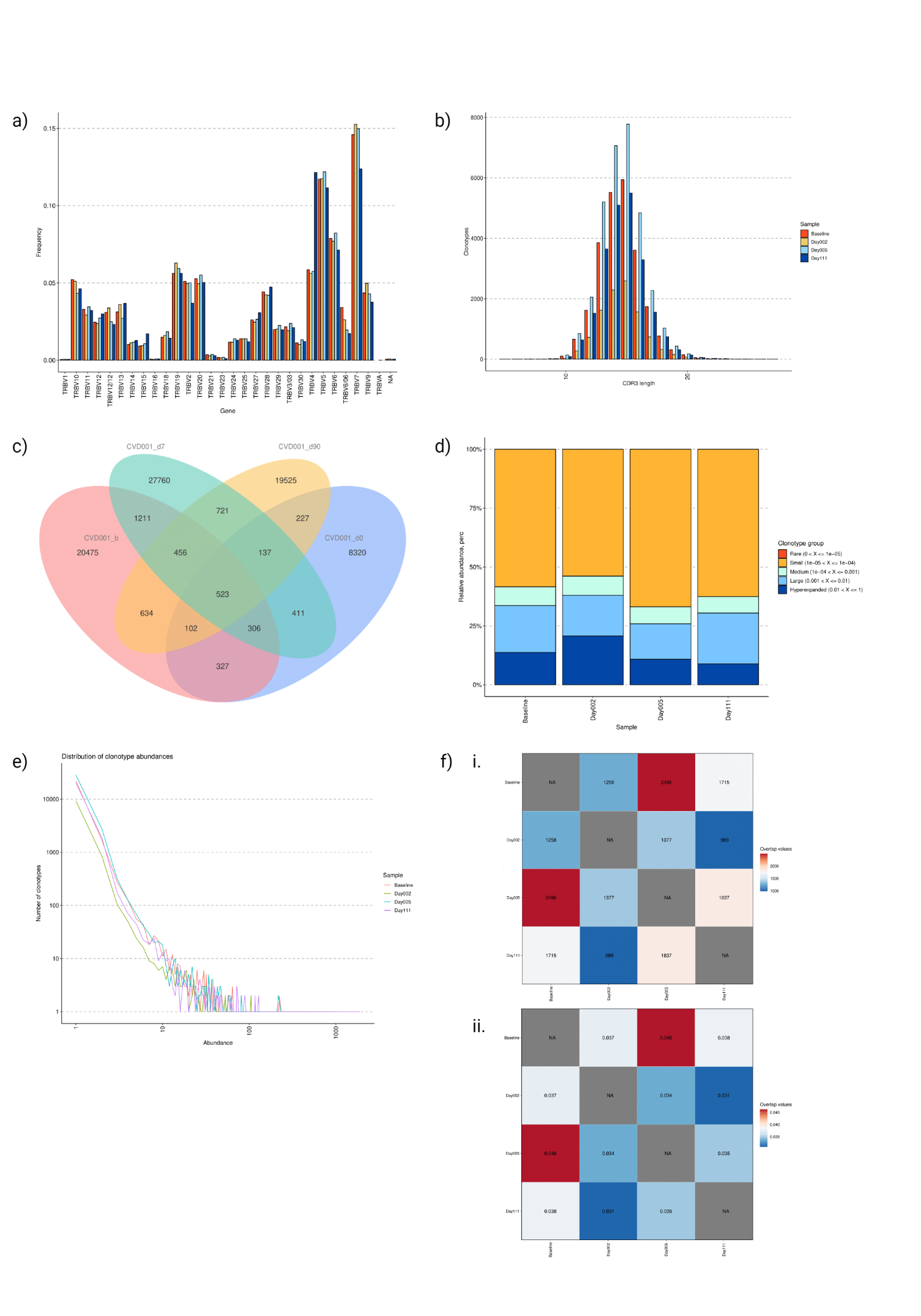

Supplement: Supplementary file 1 [file DataSheet_1.docx]
